# Associations of racial and ethnic discrimination with adverse changes in exercise and screen time during the COVID-19 pandemic in the United States

**DOI:** 10.4178/epih.e2023013

**Published:** 2023-01-28

**Authors:** Tong Xia, Gilbert C. Gee, Jian Li, Xinyue Liu, Jin Dai, Lu Shi, Donglan Zhang, Zhuo Chen, Xuesong Han, Yan Li, Hongmei Li, Ming Wen, Dejun Su, Liwei Chen

**Affiliations:** 1 Department of Epidemiology, Fielding School of Public Health, University of California Los Angeles, Los Angeles, CA, USA; 2 Department of Community Health Sciences, Fielding School of Public Health, University of California Los Angeles, Los Angeles, CA, USA; 3 Department of Environmental Health Sciences, Fielding School of Public Health, University of California Los Angeles, Los Angeles, CA, USA; 4 School of Nursing, University of California Los Angeles, Los Angeles, CA, USA; 5 Department of Public Health Sciences, Clemson University, Clemson, SC, USA; 6 Division of Health Services Research, Department of Foundations of Medicine, New York University Long Island School of Medicine, Mineola, NY, USA; 7 Department of Health Policy and Management, College of Public Health, University of Georgia, Athens, GA, USA; 8 School of Economics, Faculty of Humanities and Social Science, University of Nottingham Ningbo China, Ningbo, China; 9 Surveillance and Health Equity Science, American Cancer Society, Atlanta, GA, USA; 10 Department of Population Health Science and Policy, Icahn School of Medicine at Mount Sinai, New York, NY, USA; 11 Department of Media, Journalism and Film, Miami University, Oxford, OH, USA; 12 Department of Sociology, University of Utah, Salt Lake City, UT, USA; 13 Department of Sociology, Faculty of Social Sciences, University of Hong Kong, Hong Kong; 14 Department of Health Promotion, College of Public Health, University of Nebraska Medical Center, Omaha, NE, USA

**Keywords:** COVID-19, Racial and ethnic discrimination, Exercise, Screen time, Life style

## Abstract

**OBJECTIVES:**

During the coronavirus disease 2019 (COVID-19) pandemic, a growing prevalence of racial and ethnic discrimination occurred when many Americans struggled to maintain healthy lifestyles. This study investigated the associations of racial and ethnic discrimination with changes in exercise and screen time during the pandemic in the United States.

**METHODS:**

We included 2,613 adults who self-identified as non-Hispanic White, non-Hispanic Black, non-Hispanic Asian, or Hispanic from the Health, Ethnicity, and Pandemic study, a cross-sectional survey conducted among a nationally representative sample of United States adults between October and November 2020. We assessed self-reported racial and ethnic discrimination by measuring COVID-19-related racial and ethnic bias and examined its associations with changes in exercise and screen time using multivariable logistic regression models. We analyzed data between September 2021 and March 2022.

**RESULTS:**

COVID-19-related racial and ethnic bias was associated with decreased exercise time among non-Hispanic Asian (odds ratio [OR], 1.46; 95% confidence interval [CI], 1.13 to 1.89) and Hispanic people (OR, 1.91; 95% CI, 1.32 to 2.77), and with increased screen time among non-Hispanic Black people (OR, 1.94; 95% CI, 1.33 to 2.85), adjusting for age, sex, education, marital status, annual household income, insurance, and employment status.

**CONCLUSIONS:**

Racial and ethnic discrimination may have adversely influenced exercise and screen time changes among racial and ethnic minorities during the COVID-19 pandemic in the United States. Further studies are needed to investigate the mechanisms through which racial and ethnic discrimination can impact lifestyles and to develop potential strategies to address racial and ethnic discrimination as a barrier to healthy lifestyles.

## GRAPHICAL ABSTRACT

[Fig f3-epih-45-e2023013]

## INTRODUCTION

Beginning in mid-March 2020, many states in the United States announced stay-at-home orders to reduce the transmission of coronavirus disease 2019 (COVID-19) [1,2]. Although these restrictions were necessary to attenuate the spread of the disease, they increased social isolation, which may result in undesired behavior and lifestyle changes, such as less physical activity and more sedentary lifestyles [3-5], leading to adverse health outcomes [6,7].

Racial and ethnic discrimination in the United States is a deeply-rooted problem that induces racial inequality in social (e.g., employment and housing) [8] and health domains (e.g., depression, anxiety, hypertension, and cardiovascular disease) [9,10]. Additionally, when racial and ethnic minority groups are perceived as being associated with disease outbreaks (i.e., Black [Ebola], Asian [SARS, COVID-19], or Hispanic [H1N1], they often experience more discrimination than White people [11]. Consequently, aggravated discrimination against Asian people and Black people has been reported during the COVID-19 pandemic [11,12].

Before the pandemic, racial and ethnic discrimination had been linked to unhealthy lifestyles. Specifically, racial and ethnic discrimination has been associated with more cigarette smoking [9,13,14] and poor eating habits [15] in Blacks, Asians, Hispanics, and Whites, more alcohol drinking [14,16,17] and substance use [10,16,17] in Blacks, Asians and Hispanics, and more screen time [18] in Blacks and Whites. During the pandemic, the prevalence of racial and ethnic discrimination increased [11,12], and maintaining healthy lifestyles became challenging [3-5,19,20]. Indeed, a Canadian study found that during the pandemic, racial and ethnic discrimination was associated with decreased exercise among non-Indigenous and non-White individuals [21]. However, no studies have assessed the associations of racial and ethnic discrimination with exercise and screen time during the pandemic in the United States. Furthermore, different racial and ethnic groups may have different resilience to discrimination [22]. Meanwhile, the prevalence of racial and ethnic discrimination [12] and decreased exercise and increased screen time [3] varied across racial and ethnic groups. Thus, this study aimed to examine whether racial and ethnic discrimination was associated with changes in exercise and screen time during the COVID-19 pandemic in racial and ethnic groups using a nationally representative, population-based sample.

## MATERIALS AND METHODS

### Study design and population

The participants were selected from the cross-sectional Health, Ethnicity, and Pandemic (HEAP) study, which included a nationally representative sample (n = 2,709) of American adults aged ≥ 18 years. The HEAP study was conducted by the National Opinion Research Center (NORC) at the University of Chicago and led by the Center for Reducing Health Disparities at the University of Nebraska Medical Center between October and November 2020. A strength of this study is that it oversampled non-Hispanic Asian people (n= 977) and other minority groups. The unique sample of the HEAP study enables adequate investigations of changes in health behaviors among racial minorities and assessments of their associations with racial and ethnic discrimination during the pandemic. More details on the HEAP study design have been published [3,23,24]. We excluded participants who did not report race and ethnicity (n=3) and participants (n=93) whose self-reported race and ethnicity was not non-Hispanic White, nonHispanic Black, non-Hispanic Asian, or Hispanic due to the small sample size (i.e., American Indian or Alaska Native: n= 7, Pacific Islander: n= 35, multiracial: n= 34, other: n= 17). Finally, 2,613 participants were included in this analysis (Supplementary Material 1). The analysis was conducted between September 2021 and March 2022. This manuscript was written following the Strengthening the Reporting of Observational Studies in Epidemiology (STROBE) guidelines.

### Assessment of racial discrimination

We assessed self-reported racial and ethnic discrimination by measuring COVID-19-related racial and ethnic bias through the 9-item Coronavirus Racial Bias Scale (CRBS), which demonstrated high internal consistency reliability (Cronbach’s alpha= 0.89) [25]. The CRBS asked about beliefs on how the COVID-19 pandemic negatively affected societal attitudes toward one’s own race/ ethnicity. Sample statements in the CRBS include “I believe the country has become more dangerous for people in my racial/ethnic group because of the coronavirus”; “People of my race/ethnicity are more likely to lose their job because of the coronavirus”, and so on. Response scales ranged from 1 (strongly disagree) to 4 (strongly agree). We calculated the CRBS by averaging scores of the 9 items.

### Assessment of exercise and screen time changes, and covariates

We assessed exercise and screen time changes through two questions that asked participants to report those behaviors before and during the pandemic: number of minutes spent on physical exercises (e.g., running, walking, swimming, sports, yoga, strength training) each day; and number of hours spent on screen time (e.g., TV, computer, cellphone, iPad, etc.) each day. We calculated changes in exercise and screen time as differences between time spent on these behaviors during the pandemic and before the pandemic, and classified participants into groups according to their changes. We classified changes in exercise time as “decreased” (i.e., change in exercise time < 0 minutes) versus “not decreased” (i.e., change in exercise time ≥ 0 minutes). Changes in screen time were classified as “increased” (i.e., change in screen time > 0 hours) versus “not increased” (i.e., change in screen time ≤ 0 hours). We also collected demographic variables, including age, sex, education, marital status, annual household income, insurance, employment status before the pandemic, and geographic region in the HEAP survey.

### Statistical analysis

We applied weights to the analyses due to the sampling procedure and survey design. We used SAS version 9.4 (SAS Institute Inc., Cary, NC, USA) and considered the statistically significant level as a two-sided α level < 0.05.

We described the characteristics of participants and the distribution of racial and ethnic discrimination and lifestyle changes. The weighted percentage and actual frequency (% [n]) were reported for categorical variables and the weighted mean and standard error (SE) were reported for continuous variables. We used the chi-square test for categorical variables and one-way analysis of variance for continuous variables to make comparisons across the four racial and ethnic groups. We also used the chi-square test for categorical variables and the t-test for continuous variables to compare each racial and ethnic minority group to non-Hispanic White people. We applied the Wilcoxon signed-rank test to compare exercise and screen time before and during the pandemic in each racial and ethnic group. The missing values were 2.5% (n= 65), 3.3% (n= 87), 3.4% (n= 90), 0.7% (n= 19), and 0.2% (n= 6) for the CRBS, changes of exercise, changes in screen time, insurance, and employment status before the pandemic, respectively.

For the primary analysis, we examined the association of COVID-19-related racial and ethnic bias with decreased exercise time (yes vs. no) or increased screen time (yes vs. no) in each racial and ethnic group. We used logistic regression models with weights, with and without adjusting for potential confounders, including age (18-29, 30-44, 45-59, or ≥ 60 years), sex (male or female), marital status (widowed/divorced/separated, never married, or married/living with a partner), education (high school or less, associates, or a bachelor’s or higher degree), annual household income (< US$25,000, 25,000-49,999, or ≥ 50,000), insurance (uninsured, Medicare, Medicaid or other, or private insurance), and employment status before the pandemic (yes, no, or students/retirees). For sensitivity analyses, we examined the associations of COVID-19-related racial and ethnic bias with continuous lifestyle changes during the pandemic using multivariable linear regression models with weights adjusting for the same potential confounders as the primary analysis. We also examined the associations of COVID-19-related racial and ethnic bias with more extreme exercise time changes (decreased exercise time ≥ 30 min/day vs. increased exercise time ≥ 30 min/day). In addition, we conducted the above primary analyses using multivariable logistic regression models without applying the weights. Since previous studies have shown that the prevalence of racial and ethnic discrimination [26,27] and unhealthy behaviors [28,29] in the United States might be different by geographic region, we further examined the associations of COVID-19-related racial and ethnic bias with lifestyle changes, stratified by geographic region (i.e., Northwest, Midwest, South, and West). We calculated p-values for the interaction effect of discrimination and region on changes in exercise and screen time using the Wald chi-square test.

### Ethics statement

The Institutional Review Board of the National Opinion Research Center (NORC), University of Chicago approved the study (NORC IRB protocol #20.10.43). All participants provided written informed consent.

## RESULTS

### Characteristics of study participants

The racial/ethnic and sex distributions (weighted %) of the study sample were similar to those of the United States adult population (i.e., 63.8% non-Hispanic White people, 17.3% Hispanic people, 12.4% non-Hispanic Black people, and 6.5% non-Hispanic Asian people; 51.5% females). Among four racial/ethnic groups, non-Hispanic White people (36.0%) had the highest percentage of age ≥ 60 years. Hispanic people (57.4%) had the highest percentage of high school or less degree, and non-Hispanic Asian people (57.4%) had the highest percentage of bachelor’s or higher degree. NonHispanic Black people had the highest percentages of being never married (42.0%), having annual household income < $25,000 (35.2%), having Medicaid or other insurance (30.3%), and living in the South (58.7%), as well as the lowest percentage of living in the West (9.1%). Hispanic people (19.7%) had the highest percentage of not being employed before the pandemic (Table 1).

### Racial and ethnic discrimination and changes in exercise and screen time

**Figure 1.**
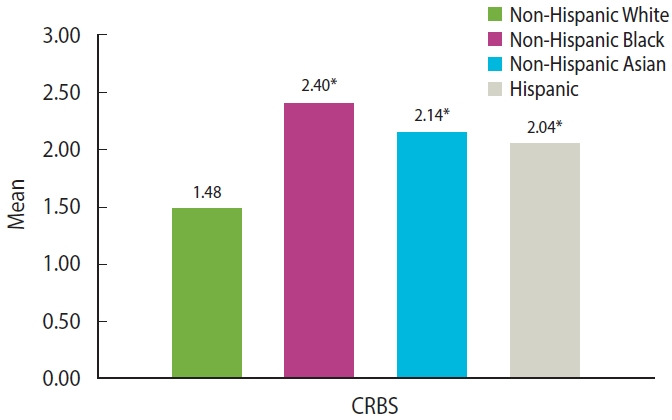
Mean values of the Coronavirus Racial Bias Scale (CRBS) by racial/ethnic groups during the coronavirus disease 2019 (COVID-19) pandemic in the Health, Ethnicity, and Pandemic study. The 9-item CRBS assessed beliefs about how the coronavirus has affected people’s race/ethnicity. Response scales ranged from 1 (strongly disagree) to 4 (strongly agree). We calculated the CRBS by adding and averaging the scores of the 9 items. ^*^p<0.05 when comparing the three racial minorities to Non-Hispanic White people using the t-test.

During the pandemic, non-Hispanic Black people had the highest score of COVID-19-related racial and ethnic bias (2.40 [0.04]), followed by non-Hispanic Asian people (2.14 [0.03]), and Hispanic people (2.04 [0.04]). The scores in these three racial/ethnic groups were higher than in non-Hispanic White people (1.48 [0.03]) (Figure 1). Meanwhile, the scores of all 9 items for calculating the COVID-19-related racial and ethnic bias were also higher in these three racial and ethnic groups than in non-Hispanic White people (Supplementary Material 2).

**Figure 2.**
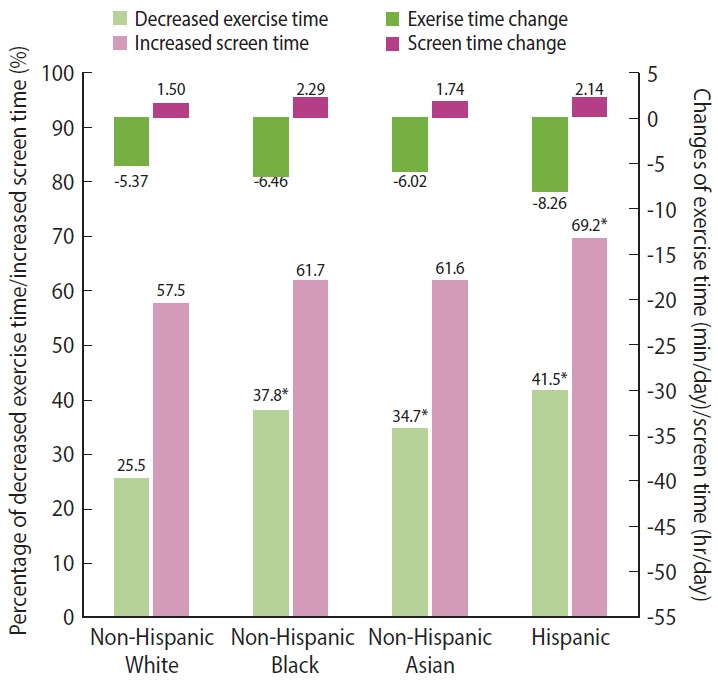
Lifestyle changes by racial/ethnic groups during the coronavirus disease 2019 pandemic in the Health, Ethnicity, and Pandemic study. For mean values of continuous changes in exercise time (min/day) and screen time (hr/day), changes were defined as the time spent on a given lifestyle factor during the pandemic minus the corresponding time before the pandemic. ^*^p<0.05 when comparing the three racial minorities to Non-Hispanic White people using the chi-square test.

Compared to the pre-pandemic, all racial/ethnic groups showed decreased exercise time (non-Hispanic White: mean [SE] of the difference between exercise time during and before the pandemic [min/day], -5.37 [1.73]; non-Hispanic Black: -6.46 [2.32]; non-Hispanic Asian: -6.02 [1.41]; Hispanic: -8.26 [2.67]) and increased screen time (non-Hispanic White: mean [SE] of the difference between screen time during and before the pandemic [hr/day], 1.50 [0.11]; non-Hispanic Black: 2.29 [0.17]; non-Hispanic Asian: 1.74 [0.11]; Hispanic: 2.14 [0.24]) during the pandemic. Compared to non-Hispanic White people (25.5%), non-Hispanic Black people (37.8%), non-Hispanic Asian people (34.7%), and Hispanic people (41.5%) had a significantly higher percentage of decreased exercise time. For screen time, only Hispanic people (69.2%) had a significantly higher percentage of increased screen time than non-Hispanic White people (57.5%) (Figure 2).

### Associations of racial and ethnic discrimination with changes in exercise and screen time

We reported unadjusted and adjusted associations of racial and ethnic discrimination with changes in exercise and screen time in Table 2. In unadjusted models, COVID-19-related racial and ethnic bias was associated with decreased exercise time among nonHispanic Asian people and Hispanic people, and with increased screen time among non-Hispanic Black people and non-Hispanic Asian people. After adjusting for age, sex, marital status, education, annual household income, insurance, and employment status, the association of COVID-19-related racial and ethnic bias with decreased exercise time remained statistically significant among nonHispanic Asian people (odds ratio [OR], 1.46; 95% confidence interval [CI], 1.13 to 1.89; p= 0.004) and Hispanic people (OR, 1.91; 95% CI, 1.32 to 2.77; p= 0.001), but the association between COVID-19-related racial and ethnic bias and increased screen time was only statistically significant among non-Hispanic Black people (OR, 1.94; 95% CI, 1.33 to 2.85; p= 0.001). In sensitivity analyses, using continuous exercise and screen time changes as the outcome, we found similar patterns of associations (Supplementary Material 3). When using larger changes of exercise (decreased exercise time ≥ 30 vs. increased exercise time ≥ 30 min/day) as the outcome, we also found similar patterns of associations, and the associations were stronger (Supplementary Material 4). Additionally, when the analyses were conducted without applying weights, the results remained the same (Supplementary Material 5).

When stratified by geographic region, the positive association between COVID-19-related racial and ethnic bias and increased screen time among non-Hispanic Black people was stronger and statistically significant in the South (OR, 3.49; 95% CI, 1.90 to 6.41; p< 0.001). The test for interaction was borderline significant (p for interaction= 0.060) (Supplementary Material 6).

## DISCUSSION

In this cross-sectional study with nationally representative data, we found that racial and ethnic discrimination was associated with unfavorable changes in exercise and screen time among racial and ethnic minorities during the COVID-19 pandemic in the United States, independent of age, sex, marital status, education, annual household income, insurance, and employment status. Specifically, self-reported COVID-19-related racial and ethnic bias was associated with decreased exercise time among non-Hispanic Asian people and Hispanic people, and with increased screen time among non-Hispanic Black people.

The reported score for COVID-19-related racial and ethnic bias was higher among Asians, Hispanics, and Blacks than among Whites. Research has noted that, although much of the discrimination related to COVID-19 was targeted toward Asians, there were spillover effects on Hispanics due to a generalized distrust of immigrant communities [30,31]. The extensively reported COVID-19-related discrimination against Blacks may have been due to the unjust police killings of several Black people at the early stage of the pandemic (e.g., George Floyd, Ahmaud Arbery, and Breyonna Taylor). Previous research has also suggested that Asians are more likely to under-report discrimination due to concerns that their experiences may be dismissed or not attribute unfair actions to discrimination [32]. All of these issues might have partially driven the patterns of reporting discrimination in our study.

This is the first study to assess the associations of racial and ethnic discrimination with exercise and screen time changes during the pandemic in the United States. Our findings on decreased exercise time are consistent with another cross-sectional study in Canada, which reported that racial and ethnic discrimination was associated with decreased exercise (OR, 2.18; 95% CI, 1.05 to 4.52) among 210 visible minorities (i.e., persons who were non-Indigenous and non-White) during the pandemic [21]. Our findings of the positive association between racial and ethnic discrimination and increased screen time also align with a cross-sectional study before the pandemic using data from the United States Coronary Artery Risk Development in Young Adults (CARDIA) study among 1,505 Black individuals and 1,765 White individuals [18].

Discrimination was related to decreased exercise time among Asian and Hispanic people, even after controlling for factors such as household income and pre-pandemic employment. A plausible reason for this may be due to fears of experiencing a hate incident. A national report from Stop AAPI Hate (where “AAPI” denotes Asian Americans and Pacific Islanders) showed that one of the most commonly reported places where the discrimination occurred in 2020 was outside on public streets [33]. Furthermore, anecdotal reports showed that Asian seniors have avoided going outside for fear of being targeted by hate crimes [34]. Some research has also noted that, although much of the discrimination related to COVID-19 was targeted towards East Asians, there were spillover effects onto Hispanic communities (but not to Black or White communities), perhaps due to a generalized distrust of foreigners [30]. Gee et al. [31] study furnishes an excellent example of this phenomenon, as shown by a racist social media quote, “… those Mexican murderers and rapists are also at the coronavirus epicenter”. To the extent that LatinX people also felt stigma and experienced discrimination related to COVID-19, they may also have opted to minimize their time outdoors, and accordingly reduced their exercise time.

Reports of COVID-19 discrimination were also related to increased screen time among Asians and African Americans, although the association for Asians became non-significant after controlling for household income and other covariates. One possible reason for the increased screen time among African Americans might have been due to the unfortunate events related to the police killings of numerous Black persons during the pandemic, including (but not limited to) George Floyd, Ahmaud Arbery, and Breyonna Taylor. Increased screen time might have been due to increased communication and information-seeking online related to these and other important events. We did not observe an association between discrimination and reporting of decreased exercise among Black participants. We speculate that this might be due to differences in the types of exercise performed by different groups. As noted above, concerns of discrimination may discourage Asians from walking outdoors, but this type of physical activity might not be the predominant form for African Americans, and accordingly, not garner an association with discrimination.

The positive association of racial and ethnic discrimination with decreased exercise time was stronger in Hispanics than in Asians. To understand whether this can be explained by the different socioeconomic profiles between Hispanics and Asians, we compared the socioeconomic status (SES) factors that were different between the Hispanic and Asian participants (i.e., education, income, health insurance, and employment; Table 1) and examined the associations of these SES factors with exercise (Supplementary Material 7). We found that only education and health insurance were significantly associated with decreased exercise. We then conducted stratified analyses by education and health insurance and found that the association of racial discrimination with decreased exercise time was stronger in individuals who did not have private insurance and who had lower education levels in both Hispanics and Asians (Supplementary Material 8). Since the proportions of people with non-private insurance and lower education level were higher in Hispanics than in Asians, the differences in health insurance status and education level between Hispanics and Asians could be a possible explanation for this finding. Interestingly, another cross-sectional study among 90,971 United States females also found a stronger association between COVID-19-related financial stressors and undesirable behavior changes (i.e., less exercise, unhealthy eating and sleep, and more alcohol drinking and smoking) in uninsured females than in insured females (OR: 2.46 vs. 1.54) [35]. In addition, we found that the discrimination-exercise association was much stronger in Hispanics who lived in the Northeast region (OR, 6.58; Supplementary Material 6), which could also help explain the relatively stronger association in Hispanics than in Asians in our study. It is noteworthy that the interrelationships among racial discrimination, undesired health behaviors, and SES are likely more complicated. The beforementioned explanations are mainly based on our data, and we could not rule out other reasons.

Our findings stratified by geographic region should be considered preliminary, but are worth mentioning as these may could help identify more vulnerable populations by region. We found that the positive association between racial and ethnic discrimination and increased screen time among non-Hispanic Black people was stronger in the South, which may be partially explained by the distribution of sex. In our study, for non-Hispanic Black people, the region with the highest percentage of females (60.5%) was the South. A meta-analysis showed that females might have less healthy behaviors against racial and ethnic discrimination than males [36]. Our study may not have adequate power for region-specific associations. Future studies with larger sample sizes are needed to test the effects of racial and ethnic discrimination by region and explore the reasons behind it.

### Strengths and limitations

To our knowledge, this is the first study examining the associations of racial and ethnic discrimination with exercise and screen time changes during the pandemic in the United States. Additionally, our study used a nationally representative sample that included multiple racial and ethnic groups with a large sample size for non-Hispanic Asian people and other minorities. Nonetheless, there are several limitations of our study. First, as it was a crosssectional study and the data were only collected at one time point during the pandemic, the findings may not reflect the associations during the later stage of the pandemic. Future studies with longitudinal designs are warranted to confirm these findings. Second, because of the observational study design, even though we tried to adjust for potential confounders, there might have been residual confounding due to unmeasured confounders (e.g., other types of racial discrimination, such as police killings of several Black people during the pandemic). Third, all measures (e.g., racial and ethnic discrimination, and exercise and screen time) were self-reported, and the exercise and screen time before and during the pandemic were measured at one time during the pandemic; therefore, the study may have been subject to information bias. Ongoing cohort studies that objectively collect exercise and screen time information before and during the pandemic are warranted to confirm our results. Fourth, the findings of this study could not be generalized to some minority groups (e.g., American Indian or Alaska Native, Pacific Islander, or multiracial group) as these groups were excluded due to very small sample sizes.

In conclusion, in a nationally representative sample, we found that racial and ethnic discrimination was associated with decreased exercise time and increased screen time among racial and ethnic minorities during the surge of the COVID-19 pandemic in the United States, and there were racial differences in these associations. Future studies with longitudinal designs are needed to examine whether such associations are causal, and if so, to identify the specific mechanisms through which racial and ethnic discrimination can negatively impact lifestyles. It is essential to examine the associations between racial and ethnic discrimination and lifestyle determinants of health outcomes during the pandemic and to identify the most vulnerable racial and ethnic groups in order to provide evidence for public health responses. Research on potential strategies to address racial and ethnic discrimination as a barrier to healthy behaviors, particularly among vulnerable groups, is necessary to inform practices and policies in future public health crises.

## DATA AVAILABILITY

Data requests can be sent to the corresponding author and the principal investigator at the Center for Reducing Health Disparities at University of Nebraska Medical Center. Data are available after approval.

## Figures and Tables

**Figure f3-epih-45-e2023013:**
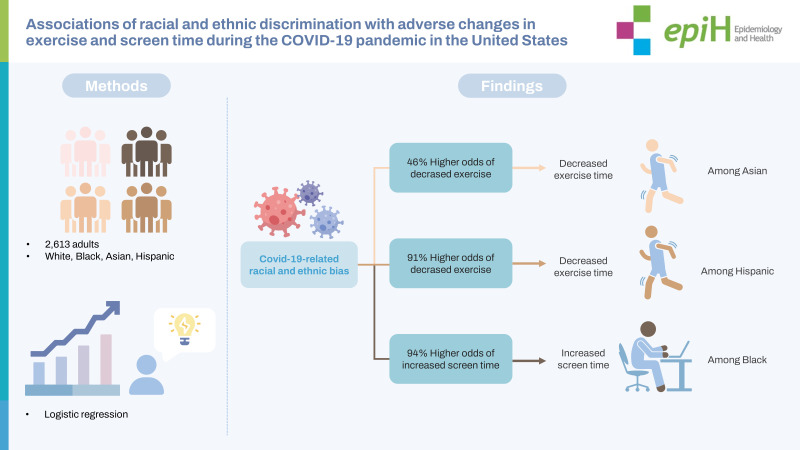


**Table 1.** Characteristics of participants by racial/ethnic groups in the HEAP study

Characteristics		Overall	Non-Hispanic White	Non-Hispanic Black	Non-Hispanic Asian	Hispanic	p-value^1^
Total		100 (2,613)	63.8 (514)	12.4 (590)	6.5 (977)	17.3 (532)	
Age (yr)							<0.001
	18-29	20.4 (545)	17.5 (81)	24.0 (97)	22.0 (228)	27.7 (139)	
	30-44	25.4 (851)	22.4 (136)	29.0 (212)	33.4 (309)	30.9 (194)	
	45-59	23.7 (548)	24.1 (96)	21.9 (131)	22.0 (200)	24.4 (121)	
	≥60	30.5 (669)	36.0 (201)	25.1 (150)	22.6 (240)	16.9 (78)	
Female		51.5 (1,359)	51.0 (223)	54.6 (340)	52.9 (562)	50.3 (234)	0.700
Education							<0.001
	High school or less	38.6 (569)	34.0 (111)	44.0 (164)	24.5 (118)	57.4 (176)	
	Associates	27.8 (1,030)	29.1 (230)	29.9 (274)	18.1 (258)	25.0 (268)	
	Bachelor’s or higher	33.6 (1,014)	37.0 (173)	26.1 (152)	57.4 (601)	17.6 (88)	
Marital status							<0.001
	Widowed/divorced/separated	17.1 (389)	17.0 (91)	20.4 (127)	11.2 (92)	17.4 (79)	
	Never married	24.4 (823)	19.3 (102)	42.0 (226)	33.1 (340)	27.2 (155)	
	Married/living with partner	58.5 (1,401)	63.7 (321)	37.6 (237)	55.7 (545)	55.4 (298)	
Annual household income (US$)							<0.001
	<25,000	19.0 (547)	13.7 (76)	35.2 (195)	15.4 (144)	28.2 (132)	
	25,000-49,999	22.7 (599)	21.6 (112)	25.1 (165)	18.9 (170)	26.5 (152)	
	≥50,000	58.3 (1,467)	64.7 (326)	39.8 (230)	65.7 (663)	45.3 (248)	
Health insurance before the pandemic							<0.001
	Uninsured	8.5 (215)	7.2 (35)	7.6 (46)	7.3 (67)	14.9 (67)	
	Medicare	21.6 (518)	23.4 (130)	17.4 (106)	20.5 (200)	18.2 (82)	
	Medicaid or other	17.8 (437)	15.1 (73)	30.3 (166)	10.9 (98)	21.5 (100)	
	Private	52.0 (1,424)	54.2 (275)	44.8 (267)	61.3 (607)	45.5 (275)	
Employment status before the pandemic							<0.001
	Yes	61.8 (1,685)	58.4 (296)	69.8 (419)	60.4 (590)	69.4 (380)	
	No	17.4 (422)	17.1 (85)	15.7 (87)	17.5 (160)	19.7 (90)	
	Students and retirees	20.8 (500)	24.5 (133)	14.4 (83)	22.1 (225)	11.0 (59)	
Geographic region							<0.001
	Northeast	17.7 (385)	18.8 (78)	15.2 (72)	21.2 (171)	14.2 (64)	
	Midwest	21.0 (461)	26.1 (154)	17.0 (145)	11.9 (108)	8.7 (54)	
	South	37.4 (870)	34.3 (153)	58.7 (315)	23.9 (213)	38.6 (189)	
	West	23.8 (897)	20.8 (129)	9.1 (58)	43.1 (485)	38.5 (225)	

Values are presented as weighted % (actual frequency), % (n) for categorical variables. HEAP, Health, Ethnicity, and Pandemic.

1 p-values were compared between four racial/ethnic groups using the chi-square test for categorical variables.

**Table 2.** Associations of COVID-19-related racial and ethnic bias^1^ with lifestyle changes during the COVID-19 pandemic^2^

Exposure: CRBS	Exercise time (decreased vs. not decreased)				Screen time (increased vs. not increased)			
	Model 1	p-value	Model 2	p-value	Model 1	p-value	Model 2	p-value
Non-Hispanic White	1.34 (0.90, 1.99)	0.150	1.25 (0.86, 1.84)	0.250	0.91 (0.63, 1.33)	0.630	0.98 (0.65, 1.47)	0.910
Non-Hispanic Black	1.10 (0.77, 1.59)	0.600	1.16 (0.78, 1.73)	0.470	1.87 (1.29, 2.69)	0.001	1.94 (1.33, 2.85)	0.001
Non-Hispanic Asian	1.56 (1.20, 2.01)	0.001	1.46 (1.13, 1.89)	0.004	1.36 (1.07, 1.72)	0.010	1.22 (0.95, 1.55)	0.110
Hispanic	1.88 (1.31, 2.71)	0.001	1.91 (1.32, 2.77)	0.001	1.28 (0.89, 1.84)	0.180	1.31 (0.91, 1.88)	0.140

Values are presented as odds ratio (95% confidence interval). COVID-19, coronavirus disease 2019; CRBS, Coronavirus Racial Bias Scale.

1 We measured COVID-19-related racial and ethnic bias using the 9-item CRBS, which assessed beliefs about how the COVID-19 pandemic affected people’s race/ethnicity; The response scales ranged from 1 (strongly disagree) to 4 (strongly agree); We calculated the CRBS by adding and averaging the scores for all 9 items.

2 Logistical regression models were used; Model 1: unadjusted models; Model 2: multivariable models adjusted for age, gender, marital status, education, annual household income, insurance, and employment status before the pandemic; Sampling weights were applied.

## SUPPLEMENTARY MATERIALS

Supplementary Material 1. Participants flowchart

Supplementary Material 2. Distribution of all 9 items in score for COVID-19-related racial and ethnic bias by racial and ethnic groups

Supplementary Material 3. Associations of COVID-19-related racial and ethnic bias with continuous changes in lifestyles before and during the COVID-19 pandemic

Supplementary Material 4. Associations of COVID-19-related racial and ethnic bias with more extreme changes in exercise time before and during the COVID-19 pandemic

Supplementary Material 5. Associations of COVID-19-related racial and ethnic bias with changes in lifestyles before and during the COVID-19 pandemic without applying sampling weights

Supplementary Material 6. Associations of self-reported COVID-19-related racial and ethnic bias with changes in lifestyles before and during the COVID-19 pandemic stratified by geographic region. Sample size for outcome decreased exercise time (Non-Hispanic Asian: n=162 in the Northeast, n=106 in the Midwest, n=204 in the South, n=467 in the West; Hispanic: n=60 in the Northeast, n=52 in the Midwest, n=168 in the South, n=205 in the West). Sample size for outcome increased screen time (Non-Hispanic Black: n=59 in the Northeast, n=128 in the Midwest, n=287 in the South, n=57 in the West). Multivariable models adjusted for age, gender, marital status, education, annual household income, insurance, and employment status before the pandemic. Sampling weights were applied. Odds ratio (OR) and 95% confidence interval (CI) were reported. ^*^ indicated P-values<0.05. // indicated axis break. CI, confidence interval; OR, odds ratio.

Supplementary Material 7. Multivariable associations of sociodemographic variables with changes in lifestyles before and during the COVID-19 pandemic

Supplementary Material 8. Associations of COVID-19-related racial and ethnic bias with changes in exercise time among Asian and Hispanic groups stratified by socioeconomic variables. And distribution of socioeconomic variables by Asian and Hispanic groups
