# Supplementary material for: Associations of racial and ethnic discrimination with adverse changes in exercise and screen time during the COVID-19 pandemic in the United States
Source: Epidemiol Health. 2023 Jan 28;45:e2023013. doi: 10.4178/epih.e2023013 (PMC10266926; doi:10.4178/epih.e2023013)
Supplement: Supplementary Material 1. — Participants flowchart [file epih-45-e2023013-Supplementary-1.docx]

**Supplementary Material 1.** Participants flowchart

HEAP, Health, Ethnicity, and Pandemic.
