# Supplementary material for: Associations of racial and ethnic discrimination with adverse changes in exercise and screen time during the COVID-19 pandemic in the United States
Source: Epidemiol Health. 2023 Jan 28;45:e2023013. doi: 10.4178/epih.e2023013 (PMC10266926; doi:10.4178/epih.e2023013)
Supplement: Supplementary Material 2. — Distribution of all 9 items in score for COVID-19-related racial and ethnic bias by racial and ethnic groups [file epih-45-e2023013-Supplementary-2.docx]

**Supplementary Material 2.** Distribution of all 9 items in score for COVID-19-related racial and ethnic bias by racial and ethnic groups

| **Items in score for COVID-19-related racial and ethnic bias** | **Overall** | | **Non-Hispanic White** | | **Non-Hispanic Black** | | | **Non-Hispanic Asian** | | | **Hispanic** | | | **Overall *P*** |
| --- | --- | --- | --- | --- | --- | --- | --- | --- | --- | --- | --- | --- | --- | --- |
|  | **mean** | **(SE)** | **mean** | **(SE)** | **mean** | **(SE)** | ***P*** | **mean** | **(SE)** | ***P*** | **mean** | **(SE)** | ***P*** |  |
| Item 1 | 1.94 | (0.04) | 1.67 | (0.05) | 2.73 | (0.05) | <0.001 | 2.50 | (0.04) | <0.001 | 2.17 | (0.06) | <0.001 | <0.001 |
| Item 2 | 1.85 | (0.03) | 1.50 | (0.04) | 2.77 | (0.06) | <0.001 | 2.14 | (0.04) | <0.001 | 2.37 | (0.06) | <0.001 | <0.001 |
| Item 3 | 1.44 | (0.02) | 1.20 | (0.03) | 1.91 | (0.05) | <0.001 | 2.14 | (0.04) | <0.001 | 1.71 | (0.05) | <0.001 | <0.001 |
| Item 4 | 1.83 | (0.03) | 1.57 | (0.04) | 2.46 | (0.05) | <0.001 | 2.31 | (0.04) | <0.001 | 2.14 | (0.06) | <0.001 | <0.001 |
| Item 5 | 1.85 | (0.03) | 1.58 | (0.04) | 2.78 | (0.05) | <0.001 | 1.78 | (0.03) | <0.001 | 2.24 | (0.06) | <0.001 | <0.001 |
| Item 6 | 1.74 | (0.03) | 1.37 | (0.04) | 2.81 | (0.05) | <0.001 | 2.02 | (0.04) | <0.001 | 2.24 | (0.06) | <0.001 | <0.001 |
| Item 7 | 1.34 | (0.03) | 1.22 | (0.03) | 1.58 | (0.05) | <0.001 | 1.67 | (0.03) | <0.001 | 1.52 | (0.05) | <0.001 | <0.001 |
| Item 8 | 1.75 | (0.03) | 1.56 | (0.05) | 2.20 | (0.05) | <0.001 | 2.27 | (0.04) | <0.001 | 1.94 | (0.06) | <0.001 | <0.001 |
| Item 9 | 1.85 | (0.03) | 1.64 | (0.05) | 2.39 | (0.05) | <0.001 | 2.44 | (0.04) | <0.001 | 2.02 | (0.06) | <0.001 | <0.001 |
| Note: Data were presented as weighted mean (standard errors, SE) for continuous variables.  P-values were compared between three racial and ethnic minorities and White people using t-tests for continuous variables. Overall P-values were compared between four racial and ethnic groups using one-way ANOVA for continuous variables.  Item 1: I believe the country has become more dangerous for people in my racial/ethnic group because of the Coronavirus.  Item 2: People of my race/ethnicity are more likely to lose their job because of the Coronavirus.  Item 3: I worry about people thinking I have the Coronavirus simply because of my race/ethnicity.  Item 4: Most social and mass media reports about the Coronavirus create bias against people of my racial/ethnic group.  Item 5: People of my race/ethnicity are more likely to get the Coronavirus.  Item 6: People of my race/ethnicity will not receive Coronavirus healthcare as good as the care received by other groups.  Item 7: Due to the Coronavirus I have been cyberbullied because of my race/ethnicity.  Item 8: Since the Coronavirus I have seen a lot more cyberbullying of people of my race/ethnicity.  Item 9: Negative social media posts against people of my race/ethnicity have increased because of the Coronavirus.  SE, standard error. | | | | | | | | | | | | | | |
