# Supplementary material for: Associations of racial and ethnic discrimination with adverse changes in exercise and screen time during the COVID-19 pandemic in the United States
Source: Epidemiol Health. 2023 Jan 28;45:e2023013. doi: 10.4178/epih.e2023013 (PMC10266926; doi:10.4178/epih.e2023013)
Supplement: Supplementary Material 3. — Associations of COVID-19-related racial and ethnic bias with continuous changes in lifestyles before and during the COVID-19 pandemic [file epih-45-e2023013-Supplementary-3.docx]

**Supplementary Material 3.** Associations of COVID-19-related racial and ethnic bias with continuous changes in lifestyles before and during the COVID-19 pandemic

|  | **Exercise time changes (minutes/day)** | | | **Screen time changes (hours/day)** | | |
| --- | --- | --- | --- | --- | --- | --- |
|  | **B** | **(SE)** | ***P*** | **B** | **(SE)** | ***P*** |
| **Exposure: Coronavirus Racial Bias Scale** | | | | | | |
| Non-Hispanic White | 0.31 | 2.82 | 0.91 | 0.01 | 0.23 | 0.96 |
| Non-Hispanic Black | 3.25 | 4.10 | 0.43 | **0.72** | **0.29** | **0.01** |
| Non-Hispanic Asian | **-5.73** | **2.07** | **0.01** | 0.29 | 0.14 | 0.05 |
| Hispanic | **-10.77** | **3.82** | **0.01** | 0.36 | 0.23 | 0.11 |
| Note: **Linear regression models were used.** Estimate B, standard error (SE), and P-value were reported. Boldface indicated statistical significance (*P*<0.05).  Multivariable models adjusted for age, gender, marital status, education, annual household income, insurance, and employment status before the pandemic. Sampling weights were applied.  We measured the COVID-19-related racial and ethnic bias through the 9-item Coronavirus Racial Bias Scale (CRBS), which assessed beliefs how the coronavirus has affected people’s race/ethnicity. Response scales ranged from 1 (strongly disagree) to 4 (strongly agree). We calculated the CRBS by adding and averaging scores of the 9 items.  CRBS, Coronavirus Racial Bias Scale; SE, standard error. | | | | | | |
