# Supplementary material for: Associations of racial and ethnic discrimination with adverse changes in exercise and screen time during the COVID-19 pandemic in the United States
Source: Epidemiol Health. 2023 Jan 28;45:e2023013. doi: 10.4178/epih.e2023013 (PMC10266926; doi:10.4178/epih.e2023013)
Supplement: Supplementary Material 4. — Associations of COVID-19-related racial and ethnic bias with more extreme changes in exercise time before and during the COVID-19 pandemic [file epih-45-e2023013-Supplementary-4.docx]

**Supplementary Material 4.** Associations of COVID-19-related racial and ethnic bias with more extreme changes in exercise time

before and during the COVID-19 pandemic

|  | **Exercise time (decreased exercise time ≥30 minutes/day vs. increased exercise time ≥30 minutes/day)** | | | |
| --- | --- | --- | --- | --- |
|  | **OR** | **95%CI** | | ***P*** |
| **Exposure: Coronavirus Racial Bias Scale** | | | | |
| Non-Hispanic White | 1.59 | (0.44, | 5.70) | 0.48 |
| Non-Hispanic Black | 0.67 | (0.33, | 1.36) | 0.26 |
| Non-Hispanic Asian | **1.78** | **(1.05,** | **3.02)** | **0.03** |
| Hispanic | **2.49** | **(1.26,** | **4.89)** | **0.01** |
| Note: Logistical regression models were used. Odds ratio (OR), 95% confidence interval (CI), and P-value were reported. Boldface indicated statistical significance (*P*<0.05).  Multivariable models adjusted for age, gender, marital status, education, annual household income, insurance, and employment status before the pandemic. Sampling weights were applied.  We measured the COVID-19-related racial and ethnic bias through the 9-item Coronavirus Racial Bias Scale (CRBS), which assessed beliefs how the coronavirus has affected people’s race/ethnicity. Response scales ranged from 1 (strongly disagree) to 4 (strongly agree). We calculated the CRBS by adding and averaging scores of the 9 items.  CI, confidence interval; CRBS, Coronavirus Racial Bias Scale; OR, odds ratio. | | | | |
