# Supplementary material for: Associations of racial and ethnic discrimination with adverse changes in exercise and screen time during the COVID-19 pandemic in the United States
Source: Epidemiol Health. 2023 Jan 28;45:e2023013. doi: 10.4178/epih.e2023013 (PMC10266926; doi:10.4178/epih.e2023013)
Supplement: Supplementary Material 5. — Associations of COVID-19-related racial and ethnic bias with changes in lifestyles before and during the COVID-19 pandemic without applying sampling weights [file epih-45-e2023013-Supplementary-5.docx]

**Supplementary Material 5.** Associations of COVID-19-related racial and ethnic bias with changes in lifestyles before and during the COVID-19 pandemic without applying sampling weights

|  | **Exercise time (decreased vs. not decreased)** | | | | **Screen time (increased vs. not increased)** | | | |
| --- | --- | --- | --- | --- | --- | --- | --- | --- |
|  | **OR** | **95% CI** | | ***P*** | **OR** | **95% CI** | | ***P*** |
| **Exposure: Coronavirus Racial Bias Scale** | | | | | | | | |
| Non-Hispanic White | 1.22 | (0.86, | 1.74) | 0.27 | 0.85 | (0.60, | 1.20) | 0.34 |
| Non-Hispanic Black | 1.29 | (0.96, | 1.75) | 0.09 | **1.90** | **(1.37,** | **2.64)** | **<0.001** |
| Non-Hispanic Asian | **1.39** | **(1.13,** | **1.72)** | **0.002** | 1.21 | (0.99, | 1.47) | 0.07 |
| Hispanic | **1.45** | **(1.09,** | **1.94)** | **0.01** | 1.23 | (0.90, | 1.67) | 0.19 |
| Note: Logistical regression models were used. Odds ratio (OR), 95% confidence interval (CI), and P-value were reported. Boldface indicated statistical significance (*P*<0.05).  Multivariable models adjusted for age, gender, marital status, education, annual household income, insurance, and employment status before the pandemic. **Sampling weights were not applied**.  We measured the COVID-19-related racial and ethnic bias through the 9-item Coronavirus Racial Bias Scale (CRBS), which assessed beliefs how the coronavirus has affected people’s race/ethnicity. Response scales ranged from 1 (strongly disagree) to 4 (strongly agree). We calculated the CRBS by adding and averaging scores of the 9 items.  CI, confidence interval; CRBS, Coronavirus Racial Bias Scale; OR, odds ratio. | | | | | | | | |
