# Supplementary material for: Associations of racial and ethnic discrimination with adverse changes in exercise and screen time during the COVID-19 pandemic in the United States
Source: Epidemiol Health. 2023 Jan 28;45:e2023013. doi: 10.4178/epih.e2023013 (PMC10266926; doi:10.4178/epih.e2023013)
Supplement: Supplementary Material 6. — Associations of self-reported COVID-19-related racial and ethnic bias with changes in lifestyles before and during the COVID-19 pandemic stratified by geographic region. Sample size for outcome decreased exercise time (Non-Hispanic Asian: n=162 in the Northeast, n=106 in the Midwest, n=204 in the South, n=467 in the West; Hispanic: n=60 in the Northeast, n=52 in the Midwest, n=168 in the South, n=205 in the West). Sample size for outcome increased screen time (Non-Hispanic Black: n=59 in the Northeast, n=128 in the Midwest, n=287 in the South, n=57 in the West). Multivariable models adjusted for age, gender, marital status, education, annual household income, insurance, and employment status before the pandemic. Sampling weights were applied. Odds ratio (OR) and 95% confidence interval (CI) were reported. * indicated P-values<0.05. // indicated axis break. CI, confidence interval; OR, odds ratio. [file epih-45-e2023013-Supplementary-6.docx]

**Supplementary Material 6.** Associations of self-reported COVID-19-related racial and ethnic bias with changes in lifestyles before and during the COVID-19 pandemic stratified by geographic region. Sample size for outcome decreased exercise time (Non-Hispanic Asian: n=162 in the Northeast, n=106 in the Midwest, n=204 in the South, n=467 in the West; Hispanic: n=60 in the Northeast, n=52 in the Midwest, n=168 in the South, n=205 in the West). Sample size for outcome increased screen time (Non-Hispanic Black: n=59 in the Northeast, n=128 in the Midwest, n=287 in the South, n=57 in the West). Multivariable models adjusted for age, gender, marital status, education, annual household income, insurance, and employment status before the pandemic. Sampling weights were applied. Odds ratio (OR) and 95% confidence interval (CI) were reported. * indicated P-values<0.05. // indicated axis break.

CI, confidence interval; OR, odds ratio.
