# Supplementary material for: Associations of racial and ethnic discrimination with adverse changes in exercise and screen time during the COVID-19 pandemic in the United States
Source: Epidemiol Health. 2023 Jan 28;45:e2023013. doi: 10.4178/epih.e2023013 (PMC10266926; doi:10.4178/epih.e2023013)
Supplement: Supplementary Material 7. — Multivariable associations of sociodemographic variables with changes in lifestyles before and during the COVID-19 pandemic [file epih-45-e2023013-Supplementary-7.docx]

**Supplementary Material 7.** Multivariable associations of sociodemographic variables with changes in lifestyles before and during the COVID-19 pandemic

| **Characteristics** | **Exercise time (decreased vs. not decreased)** | | | | | | | | | | | | | | | | | | | |
| --- | --- | --- | --- | --- | --- | --- | --- | --- | --- | --- | --- | --- | --- | --- | --- | --- | --- | --- | --- | --- |
|  | **Overall** | | | | **Non-Hispanic White** | | | | **Non-Hispanic Black** | | | | **Non-Hispanic Asian** | | | | **Hispanic** | | | |
|  | **OR** | **95%CI** | | ***P*** | **OR** | **95%CI** | | ***P*** | **OR** | **95%CI** | | ***P*** | **OR** | **95%CI** | | ***P*** | **OR** | **95%CI** | | ***P*** |
| Age, years |  |  |  |  |  |  |  |  |  |  |  |  |  |  |  |  |  |  |  |  |
| 18-29 | 1.15 | (0.68, | 1.96) | 0.60 | 0.97 | (0.39, | 2.46) | 0.96 | 1.63 | (0.74, | 3.61) | 0.23 | 1.54 | (0.80, | 2.97) | 0.20 | 0.73 | (0.26, | 2.05) | 0.55 |
| 30-44 | **1.87** | **(1.14,** | **3.06)** | **0.01** | 2.18 | (0.99, | 4.80) | 0.05 | 1.72 | (0.89, | 3.34) | 0.11 | 0.97 | (0.54, | 1.77) | 0.93 | 1.39 | (0.58, | 3.37) | 0.46 |
| 45-59 | 1.15 | (0.68, | 1.93) | 0.60 | 1.14 | (0.50, | 2.58) | 0.75 | 1.12 | (0.56, | 2.21) | 0.75 | 1.20 | (0.64, | 2.26) | 0.57 | 1.13 | (0.47, | 2.73) | 0.78 |
| 60+ | Ref |  |  |  | Ref |  |  |  | Ref |  |  |  | Ref |  |  |  | Ref |  |  |  |
| Gender |  |  |  |  |  |  |  |  |  |  |  |  |  |  |  |  |  |  |  |  |
| Male | Ref |  |  |  | Ref |  |  |  | Ref |  |  |  | Ref |  |  |  | Ref |  |  |  |
| Female | 1.17 | (0.87, | 1.58) | 0.29 | 0.90 | (0.56, | 1.44) | 0.65 | 1.37 | (0.87, | 2.16) | 0.17 | 1.21 | (0.84, | 1.73) | 0.30 | **2.38** | **(1.43,** | **3.96)** | **0.001** |
| Race/ethnicity |  |  |  |  |  |  |  |  |  |  |  |  |  |  |  |  |  |  |  |  |
| Non-Hispanic White | Ref |  |  |  | N/A |  |  |  | N/A |  |  |  | N/A |  |  |  | N/A |  |  |  |
| Non-Hispanic Black | 1.31 | (0.89, | 1.93) | 0.17 |  |  |  |  |  |  |  |  |  |  |  |  |  |  |  |  |
| Non-Hispanic Asian | 1.11 | (0.79, | 1.56) | 0.55 |  |  |  |  |  |  |  |  |  |  |  |  |  |  |  |  |
| Hispanic | **2.02** | **(1.41,** | **2.89)** | **<0.001** |  |  |  |  |  |  |  |  |  |  |  |  |  |  |  |  |
| Marital Status |  |  |  |  |  |  |  |  |  |  |  |  |  |  |  |  |  |  |  |  |
| Married/living with partner | Ref |  |  |  | Ref |  |  |  | Ref |  |  |  | Ref |  |  |  | Ref |  |  |  |
| Widowed/Divorced/Separated | 1.15 | (0.75, | 1.78) | 0.52 | 1.31 | (0.66, | 2.58) | 0.44 | 1.32 | (0.72, | 2.45) | 0.37 | 0.75 | (0.38, | 1.47) | 0.40 | 0.72 | (0.33, | 1.58) | 0.42 |
| Never married | 1.34 | (0.94, | 1.91) | 0.11 | 1.22 | (0.66, | 2.28) | 0.52 | 1.24 | (0.71, | 2.18) | 0.45 | 1.07 | (0.69, | 1.66) | 0.76 | **2.03** | **(1.04,** | **3.95)** | **0.04** |
| Education |  |  |  |  |  |  |  |  |  |  |  |  |  |  |  |  |  |  |  |  |
| High school or less | Ref |  |  |  | Ref |  |  |  | Ref |  |  |  | Ref |  |  |  | Ref |  |  |  |
| Associates | **1.70** | **(1.19,** | **2.43)** | **0.004** | **2.56** | **(1.30,** | **5.06)** | **0.01** | 1.62 | (0.97, | 2.69) | 0.06 | 0.76 | (0.42, | 1.36) | 0.35 | 0.83 | (0.47, | 1.45) | 0.51 |
| Bachelor’s or higher | **1.96** | **(1.30,** | **2.96)** | **0.001** | **3.25** | **(1.57,** | **6.73)** | **0.002** | 0.92 | (0.49, | 1.73) | 0.80 | 1.01 | (0.58, | 1.76) | 0.97 | 0.71 | (0.35, | 1.45) | 0.34 |
| Annual household income |  |  |  |  |  |  |  |  |  |  |  |  |  |  |  |  |  |  |  |  |
| <$25,000 | Ref |  |  |  | Ref |  |  |  | Ref |  |  |  | Ref |  |  |  | Ref |  |  |  |
| $25,000–$49,999 | 0.97 | (0.64, | 1.47) | 0.87 | 0.79 | (0.37, | 1.70) | 0.54 | 1.48 | (0.82, | 2.66) | 0.19 | 1.02 | (0.53, | 1.97) | 0.95 | 1.13 | (0.56, | 2.28) | 0.73 |
| ≥$50,000 | 0.96 | (0.63, | 1.47) | 0.86 | 0.90 | (0.41, | 1.95) | 0.78 | 1.35 | (0.70, | 2.61) | 0.37 | 0.87 | (0.47, | 1.59) | 0.64 | 1.04 | (0.49, | 2.21) | 0.91 |
| Insurance before the COVID pandemic |  |  |  |  |  |  |  |  |  |  |  |  |  |  |  |  |  |  |  |  |
| Private insurance | Ref |  |  |  | Ref |  |  |  | Ref |  |  |  | Ref |  |  |  | Ref |  |  |  |
| Uninsured | 0.88 | (0.51, | 1.52) | 0.66 | 0.75 | (0.28, | 2.02) | 0.57 | 0.88 | (0.36, | 2.15) | 0.77 | 0.66 | (0.31, | 1.41) | 0.28 | 0.85 | (0.36, | 2.01) | 0.71 |
| Medicare | **1.64** | **(1.02,** | **2.64)** | **0.04** | 1.70 | (0.76, | 3.83) | 0.20 | 1.17 | (0.57, | 2.43) | 0.67 | 1.48 | (0.86, | 2.54) | 0.15 | 1.43 | (0.61, | 3.35) | 0.41 |
| Medicaid, other | 1.33 | (0.86, | 2.06) | 0.20 | 1.53 | (0.71, | 3.29) | 0.27 | 1.07 | (0.58, | 1.95) | 0.84 | **1.89** | **(1.02,** | **3.49)** | **0.04** | 1.04 | (0.50, | 2.15) | 0.92 |
| Employment status before the COVID pandemic |  |  |  |  |  |  |  |  |  |  |  |  |  |  |  |  |  |  |  |  |
| Yes | Ref |  |  |  | Ref |  |  |  | Ref |  |  |  | Ref |  |  |  | Ref |  |  |  |
| No | 0.92 | (0.59, | 1.42) | 0.71 | 0.89 | (0.42, | 1.89) | 0.75 | 1.32 | (0.71, | 2.47) | 0.38 | 1.12 | (0.68, | 1.86) | 0.65 | 0.72 | (0.33, | 1.57) | 0.41 |
| Students and retirees | 1.11 | (0.70, | 1.76) | 0.65 | 1.23 | (0.62, | 2.45) | 0.55 | 0.76 | (0.37, | 1.55) | 0.44 | 1.16 | (0.72, | 1.87) | 0.55 | 0.79 | (0.35, | 1.80) | 0.57 |
| CRBS score | **1.39** | **(1.12,** | **1.73)** | **0.003** | 1.25 | (0.86, | 1.84) | 0.25 | 1.16 | (0.78, | 1.73) | 0.47 | **1.46** | **(1.13,** | **1.89)** | **0.004** | **1.91** | **(1.32,** | **2.77)** | **0.001** |
| **Characteristics** | **Screen time (increased vs. not increased)** | | | | | | | | | | | | | | | | | | | |
|  | **Overall** | | | | **Non-Hispanic White** | | | | **Non-Hispanic Black** | | | | **Non-Hispanic Asian** | | | | **Hispanic** | | | |
|  | **OR** | **95%CI** | | ***P*** | **OR** | **95%CI** | | ***P*** | **OR** | **95%CI** | | ***P*** | **OR** | **95%CI** | | ***P*** | **OR** | **95%CI** | | ***P*** |
| Age, years |  |  |  |  |  |  |  |  |  |  |  |  |  |  |  |  |  |  |  |  |
| 18-29 | 1.32 | (0.76, | 2.31) | 0.32 | 1.49 | (0.63, | 3.49) | 0.36 | 1.86 | (0.78, | 4.45) | 0.16 | **2.81** | **(1.40,** | **5.63)** | **0.004** | 0.41 | (0.15, | 1.10) | 0.08 |
| 30-44 | 1.40 | (0.87, | 2.26) | 0.16 | 2.04 | (0.96, | 4.34) | 0.06 | 1.14 | (0.55, | 2.36) | 0.72 | 1.55 | (0.89, | 2.72) | 0.12 | 0.51 | (0.22, | 1.17) | 0.11 |
| 45-59 | 0.86 | (0.53, | 1.40) | 0.54 | 0.75 | (0.37, | 1.52) | 0.43 | 0.99 | (0.47, | 2.11) | 0.99 | 1.58 | (0.87, | 2.88) | 0.13 | 0.86 | (0.35, | 2.09) | 0.73 |
| 60+ | Ref |  |  |  | Ref |  |  |  | Ref |  |  |  | Ref |  |  |  | Ref |  |  |  |
| Gender |  |  |  |  |  |  |  |  |  |  |  |  |  |  |  |  |  |  |  |  |
| Male | Ref |  |  |  | Ref |  |  |  | Ref |  |  |  | Ref |  |  |  | Ref |  |  |  |
| Female | **1.76** | **(1.30,** | **2.37)** | **<0.001** | **1.86** | **(1.19,** | **2.91)** | **0.01** | **2.14** | **(1.34,** | **3.44)** | **0.002** | 1.08 | (0.77, | 1.51) | 0.66 | **2.48** | **(1.44,** | **4.29)** | **0.001** |
| Race/ethnicity |  |  |  |  |  |  |  |  |  |  |  |  |  |  |  |  |  |  |  |  |
| Non-Hispanic White | Ref |  |  |  | N/A |  |  |  | N/A |  |  |  | N/A |  |  |  | N/A |  |  |  |
| Non-Hispanic Black | 1.23 | (0.81, | 1.86) | 0.34 |  |  |  |  |  |  |  |  |  |  |  |  |  |  |  |  |
| Non-Hispanic Asian | 0.93 | (0.66, | 1.32) | 0.68 |  |  |  |  |  |  |  |  |  |  |  |  |  |  |  |  |
| Hispanic | **1.91** | **(1.29,** | **2.83)** | **0.001** |  |  |  |  |  |  |  |  |  |  |  |  |  |  |  |  |
| Marital Status |  |  |  |  |  |  |  |  |  |  |  |  |  |  |  |  |  |  |  |  |
| Married/living with partner | Ref |  |  |  | Ref |  |  |  | Ref |  |  |  | Ref |  |  |  | Ref |  |  |  |
| Widowed/Divorced/Separated | 0.76 | (0.49, | 1.16) | 0.20 | 0.67 | (0.35, | 1.28) | 0.22 | 0.96 | (0.53, | 1.72) | 0.88 | 0.99 | (0.53, | 1.83) | 0.97 | 0.92 | (0.43, | 1.95) | 0.82 |
| Never married | 1.03 | (0.69, | 1.56) | 0.87 | 0.94 | (0.47, | 1.86) | 0.85 | 0.71 | (0.40, | 1.26) | 0.24 | 1.11 | (0.72, | 1.70) | 0.64 | 1.88 | (0.94, | 3.74) | 0.07 |
| Education |  |  |  |  |  |  |  |  |  |  |  |  |  |  |  |  |  |  |  |  |
| High school or less | Ref |  |  |  | Ref |  |  |  | Ref |  |  |  | Ref |  |  |  | Ref |  |  |  |
| Associates | 1.23 | (0.85, | 1.79) | 0.27 | 1.32 | (0.74, | 2.34) | 0.35 | 1.36 | (0.80, | 2.30) | 0.25 | 0.80 | (0.43, | 1.46) | 0.46 | 1.02 | (0.59, | 1.78) | 0.95 |
| Bachelor’s or higher | **1.94** | **(1.27,** | **2.95)** | **0.002** | **2.54** | **(1.37,** | **4.72)** | **0.003** | 1.22 | (0.64, | 2.34) | 0.54 | 0.74 | (0.42, | 1.30) | 0.29 | 1.06 | (0.50, | 2.22) | 0.89 |
| Annual household income |  |  |  |  |  |  |  |  |  |  |  |  |  |  |  |  |  |  |  |  |
| <$25,000 | Ref |  |  |  | Ref |  |  |  | Ref |  |  |  | Ref |  |  |  | Ref |  |  |  |
| $25,000–$49,999 | 0.98 | (0.63, | 1.52) | 0.91 | 0.75 | (0.35, | 1.59) | 0.45 | 1.61 | (0.89, | 2.92) | 0.11 | 0.97 | (0.50, | 1.88) | 0.93 | 1.21 | (0.58, | 2.54) | 0.60 |
| ≥$50,000 | 1.32 | (0.86, | 2.03) | 0.21 | 1.13 | (0.55, | 2.32) | 0.74 | 1.77 | (0.95, | 3.33) | 0.07 | 1.02 | (0.57, | 1.81) | 0.95 | 1.89 | (0.92, | 3.87) | 0.08 |
| Insurance before the COVID pandemic |  |  |  |  |  |  |  |  |  |  |  |  |  |  |  |  |  |  |  |  |
| Private insurance | Ref |  |  |  | Ref |  |  |  | Ref |  |  |  | Ref |  |  |  | Ref |  |  |  |
| Uninsured | 1.30 | (0.70, | 2.41) | 0.40 | 1.16 | (0.42, | 3.24) | 0.78 | 1.18 | (0.46, | 3.01) | 0.73 | 0.83 | (0.39, | 1.78) | 0.64 | **3.14** | **(1.24,** | **7.97)** | **0.02** |
| Medicare | 0.92 | (0.57, | 1.47) | 0.72 | 0.71 | (0.35, | 1.44) | 0.34 | **2.39** | **(1.06,** | **5.40)** | **0.04** | 1.23 | (0.70, | 2.16) | 0.48 | 1.21 | (0.49, | 2.97) | 0.68 |
| Medicaid, other | 0.99 | (0.63, | 1.56) | 0.97 | 0.89 | (0.44, | 1.83) | 0.76 | 1.30 | (0.69, | 2.44) | 0.42 | 0.89 | (0.48, | 1.65) | 0.71 | 1.50 | (0.69, | 3.26) | 0.30 |
| Employment status before the COVID pandemic |  |  |  |  |  |  |  |  |  |  |  |  |  |  |  |  |  |  |  |  |
| Yes | Ref |  |  |  | Ref |  |  |  | Ref |  |  |  | Ref |  |  |  | Ref |  |  |  |
| No | 1.28 | (0.83, | 1.98) | 0.27 | **2.00** | **(1.03,** | **3.86)** | **0.04** | 0.96 | (0.48, | 1.90) | 0.90 | **0.61** | **(0.38,** | **0.98)** | **0.04** | **0.39** | **(0.18,** | **0.82)** | **0.01** |
| Students and retirees | 1.47 | (0.91, | 2.37) | 0.11 | **2.20** | **(1.10,** | **4.40)** | **0.03** | 0.43 | (0.19, | 0.99) | 0.05 | 0.69 | (0.42, | 1.14) | 0.15 | 0.80 | (0.31, | 2.07) | 0.64 |
| CRBS score | 1.14 | (0.87, | 1.49) | 0.33 | 0.98 | (0.65, | 1.47) | 0.91 | **1.94** | **(1.33,** | **2.85)** | **0.001** | 1.22 | (0.95, | 1.55) | 0.11 | 1.31 | (0.91, | 1.88) | 0.14 |
| Note: Logistical regression models were used. Odds ratio (OR), 95% confidence interval (CI), and P-value were reported. Boldface indicated statistical significance (P<0.05).  Multivariable models adjusted for all demographic variables (i.e., age, gender, marital status, education, annual household income, insurance, and employment status before the pandemic) and the Coronavirus Racial Bias Scale (CRBS). In overall participants, race/ethnicity was also controlled in the models. Sampling weights were applied.  We measured the COVID-19-related racial and ethnic bias through the 9-item CRBS, which assessed beliefs how the coronavirus has affected people’s race/ethnicity. Response scales ranged from 1 (strongly disagree) to 4 (strongly agree). We calculated the CRBS by adding and averaging scores of the 9 items.  CI, confidence interval; CRBS, Coronavirus Racial Bias Scale; OR, odds ratio. | | | | | | | | | | | | | | | | | | | | |
