# Supplementary material for: Associations of racial and ethnic discrimination with adverse changes in exercise and screen time during the COVID-19 pandemic in the United States
Source: Epidemiol Health. 2023 Jan 28;45:e2023013. doi: 10.4178/epih.e2023013 (PMC10266926; doi:10.4178/epih.e2023013)
Supplement: Supplementary Material 8. — Associations of COVID-19-related racial and ethnic bias with changes in exercise time among Asian and Hispanic groups stratified by socioeconomic variables. And distribution of socioeconomic variables by Asian and Hispanic groups [file epih-45-e2023013-Supplementary-8.docx]

**Supplementary Material 8.** Associations of COVID-19-related racial and ethnic bias with changes in exercise time among Asian and Hispanic groups stratified by socioeconomic variables. And distribution of socioeconomic variables by Asian and Hispanic groups

|  | **Exercise time (decreased vs. not decreased)** | | | | | | | | **Distribution of education and insurance** | | | | |
| --- | --- | --- | --- | --- | --- | --- | --- | --- | --- | --- | --- | --- | --- |
|  | **Non-Hispanic Asian** | | | | **Hispanic** | | | | **Non-Hispanic Asian** | | **Hispanic** | | ***P*** |
|  | **OR** | **95%CI** | | ***P*** | **OR** | **95%CI** | | ***P*** |  |  |  |  |  |
| Education |  |  |  |  |  |  |  |  |  |  |  |  | <0.001 |
| High school or less | 1.69 | (0.72, | 3.94) | 0.22 | **2.14** | **(1.18,** | **3.90)** | **0.01** | 24.5 | (118) | 57.4 | (176) |  |
| Associates and above | **1.49** | **(1.15,** | **1.91)** | **0.002** | **1.63** | **(1.08,** | **2.46)** | **0.02** | 75.5 | (859) | 42.6 | (356) |  |
| Health insurance before the pandemic |  |  |  |  |  |  |  |  |  |  |  |  | <0.001 |
| Private insurance | **1.50** | **(1.09,** | **2.06)** | **0.01** | **1.67** | **(1.01,** | **2.75)** | **0.04** | 61.3 | (607) | 45.5 | (275) |  |
| Not having private insurance | **1.67** | **(1.06,** | **2.63)** | **0.03** | **2.32** | **(1.28,** | **4.20)** | **0.01** | 38.7 | (365) | 54.5 | (249) |  |
| Note:  For the association of Coronavirus Racial Bias Scale with decreased exercise time among Asian and Hispanic groups stratified by education and insurance:  Logistical regression models were used. Odds ratio (OR), 95% confidence interval (CI), and P-value were reported. Boldface indicated statistical significance (*P*<0.05).  Multivariable models adjusted for age, gender, marital status, education, annual household income, insurance, and employment status before the pandemic. When stratified by one variable, that variable was not controlled in the model. Sampling weights were applied.  We measured the COVID-19-related racial and ethnic bias through the 9-item Coronavirus Racial Bias Scale (CRBS), which assessed beliefs how the coronavirus has affected people’s race/ethnicity. Response scales ranged from 1 (strongly disagree) to 4 (strongly agree). We calculated the CRBS by adding and averaging scores of the 9 items.  For the distribution of education and insurance by Asian and Hispanic groups:  Data were presented as weighted percentage and actual frequency, % (N) for categorical variables.  P-values were compared between Non-Hispanic Asian and Hispanic groups using χ2-tests for categorical variables.  CI, confidence interval; CRBS, Coronavirus Racial Bias Scale; OR, odds ratio. | | | | | | | | | | | | | |
